# Maxillary Chronic Osteomyelitis Caused by Domestic Violence: A Diagnostic Challenge

**DOI:** 10.1155/2014/930169

**Published:** 2014-12-25

**Authors:** Tamyris Inácio Oliveira, Marina Lara de Carli, Noé Vital Ribeiro Junior, Alessandro Antônio Costa Pereira, Dimitris N. Tatakis, João Adolfo Costa Hanemann

**Affiliations:** ^1^Section of Stomatology, Department of Clinic and Surgery, School of Dentistry, Federal University of Alfenas, Rua Gabriel Monteiro da Silva 700, 37130-000 Alfenas, MG, Brazil; ^2^Section of Periodontology, Department of Clinic and Surgery, School of Dentistry, Federal University of Alfenas, 37130-000 Alfenas, MG, Brazil; ^3^Section of Pathology, Institute of Biomedical Sciences, Federal University of Alfenas, 37130-000 Alfenas, MG, Brazil; ^4^Division of Periodontology, College of Dentistry, The Ohio State University, Columbus, OH 43210, USA

## Abstract

Maxillary osteomyelitis is a rare condition defined as inflammation of the bone primarily caused by odontogenic bacteria, with trauma being the second leading cause. The present report documents a rare case of maxillary osteomyelitis in a 38-year-old female who was the victim of domestic violence approximately a year prior to presentation. Intraoral examination revealed a lesion appearing as exposed bony sequestrum, with significant destruction of gingiva and alveolar mucosa in the maxillary right quadrant, accompanied by significant pain, local edema, and continued purulence. Teeth numbers 11, 12, 13, 14, and 15 were mobile, not responsive to percussion, and nonvital. Treatment included antibiotic therapy for seven days followed by total enucleation of the necrotic bone tissue and extraction of the involved teeth. Microscopic findings confirmed the clinical diagnosis of chronic suppurative osteomyelitis. Six months postoperatively, the treated area presented complete healing and there was no sign of recurrence of the lesion.

## 1. Introduction

Maxillofacial trauma is a form of injury that can affect males and females of all ages and can result in facial bone fractures, dentoalveolar trauma, and soft tissue lesions, occurring in isolation or in association with other lesions in the body [1]. The main causes of maxillofacial trauma reported in the literature are automobile accidents, sports injuries, falls, gunshots, work-related accidents, iatrogenic causes, pathologic lesions, and physical aggression (e.g., interpersonal violence, fistfights), although the prevalence of the various causes differs by country [2]. The causes and severity of maxillofacial trauma vary by population and cultural differences must be considered in order to rank the most common trauma causes [1].

According to Gassner et al. [1], males are more likely to suffer maxillofacial trauma, at a 2 : 1 ratio. Although it can occur at any age, young adults, aged 21–30 years, are the group most commonly affected by maxillofacial trauma [3]. The middle and lower third of the face are the most frequently affected sites [4]. An orally relevant consequence of maxillofacial trauma is its effect on teeth and gingiva [5], since it was noted that 49.9% of patients had facial trauma in combination with dentoalveolar trauma [1]. The most common forms of dentoalveolar trauma observed in association with maxillofacial trauma were crown fracture, root fracture, subluxation, avulsion, intrusion, and tooth concussion [1]. Dentoalveolar trauma, especially when the resulting tissue damage is extensive, can significantly impair a person's quality of life because of its impact on common daily functions (e.g., swallowing, chewing, and speaking) [6].

Maxillofacial injuries are commonly seen in victims of domestic violence [7, 8]. A woman who has maxillofacial injuries is 7.5 times more likely to be a victim of domestic violence than a woman with injuries limited to other locations [9]. The fist is the preferred mechanism of injury and the midface is the most frequently affected site. Contusions, facial lacerations, and fractures of midface are the most common types of injury induced specifically by domestic violence [7], and fractures of maxilla account for only 8.7% of cases [8].

A possible complication of maxillofacial trauma may be chronic osteomyelitis, defined as inflammation of the bone primarily caused by odontogenic bacteria; trauma is the second leading cause of chronic osteomyelitis [4]. Chronic osteomyelitis may represent the long-term sequela of untreated acute osteomyelitis or a continuing, low-grade inflammatory response, which never went through a substantial or clinically evident acute phase [10]. Although there are reports of chronic osteomyelitis in the jaws [2, 11], cases occurring in the maxilla are rare, as are extensive lesions [12, 13]. To the best of our knowledge, there are no reported cases of maxillary osteomyelitis in the context of domestic violence-associated maxillofacial trauma.

The aim of this report is to present a case of chronic suppurative osteomyelitis occurring in the maxilla of a female patient as a result of maxillofacial trauma caused by domestic violence, to emphasize the lesion characteristics (size, severity of tissue destruction), the treatment rendered, and the impact on the patient's quality of life.

## 2. Case Report

A 38-year-old female was referred (May 2011) to the stomatology clinic with a lesion on the anterior right maxilla. Her medical history was unremarkable, she denied taking any medications, and she reported being a cigarette smoker (20-pack-year exposure). Approximately a year prior to presentation, she was victim of domestic violence. Her husband had punched her in the face. The patient did not report complaints to the police and did not seek medical attention after the incident. After a few months, she noticed the appearance of purulent exudate from the attached gingiva in the area between teeth 11 and 12. Since the original appearance of the fistula, the lesion increased in size and was accompanied by significant pain, local edema, and continued purulence.

**Figure 1:**
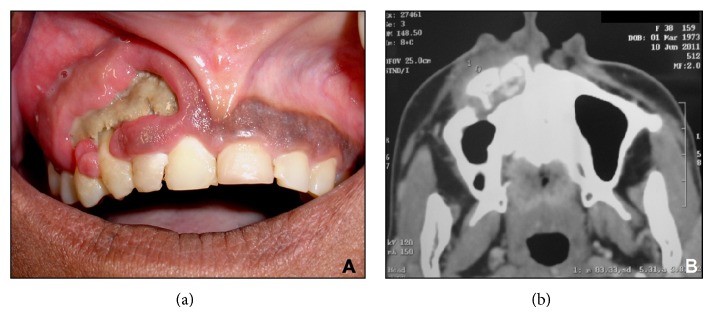
Clinical (a) and radiographic (b) image upon presentation. (a) Note the exposed bony sequestrum and significant destruction of gingiva and alveolar mucosa in the maxillary right quadrant. (b) Axial view (CT scan); note the alveolar bone destruction on the maxillary right quadrant, the surrounding radiolucent halo, and the partial destruction of the front wall of the right maxillary sinus.

Extraoral examination revealed slight facial asymmetry, caused by elevation of the right wing of the nose and edema in the midface. The overlying skin color was normal. Head and neck lymph nodes were normal to palpation. Intraoral examination revealed a lesion appearing as exposed bony sequestrum, with significant destruction of gingiva and alveolar mucosa in the maxillary right quadrant (Figure 1(a)). Edema (nontender swelling) partly obliterated the maxillary right vestibule. The corresponding palatal mucosa was also edematous and tender. Teeth 11, 12, 13, 14, and 15 were mobile, not responsive to percussion, and nonvital. Radiographic assessment (panoramic and occlusal radiographs) revealed diffuse bone destruction (“moth-eaten” appearance and vertical bone loss) in the area of teeth 11 and 12. A CT scan confirmed the bony lesion, surrounded by a radiolucent halo, causing partial destruction of the anterior wall of the right maxillary sinus (Figure 1(b)). Based on the clinical and radiographic findings, a working diagnosis of chronic suppurative osteomyelitis was made.

In addition to the lesion in the anterior maxilla, clinical and radiographic examination revealed poor dental health, with periodontal, endodontic, and restorative treatment needs.

Initial treatment consisted of oral antibiotic prescription (amoxicillin 500 mg tid and metronidazole 400 mg tid) and antimicrobial mouthwash (chlorhexidine digluconate 0.12% bid) for seven days. Following this regimen, the patient reported improvement of symptoms; however, the clinical appearance of the lesion remained unchanged. Surgical enucleation of the lesion was treatment planned and requested preoperative hematological exams were all within normal ranges.

**Figure 2:**
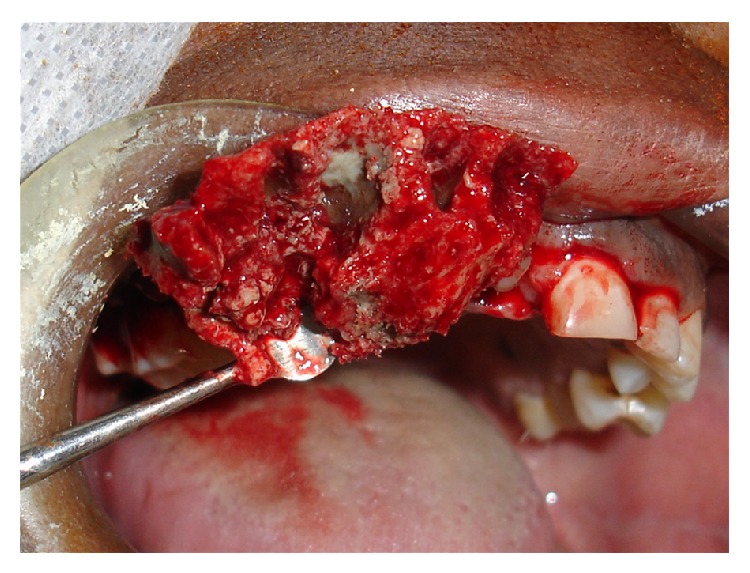
Gross specimen (sequestrum) obtained during surgery (occlusal view); note palatal and interradicular bone destruction.

The surgical intervention, performed under local anesthesia, consisted of total enucleation of the necrotic bone tissue and extraction of teeth 11, 12, 13, 14, and 15 (Figure 2). The palatal cortical bone was preserved, the surgical cavity was debrided, and bleeding was induced. The surgical cavity was subsequently filled with collagen membrane and sutured.

**Figure 3:**
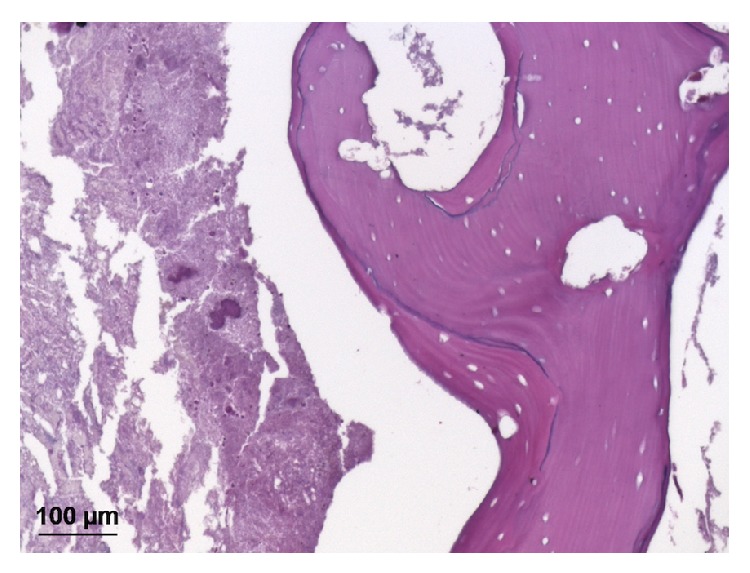
Histopathology of surgical specimen. Note the acellular and avascular osseous tissue with reversal lines and irregular surface. Interspersed between bone tissue fragments there is a slightly basophilic, amorphous, and acellular material, suggestive of microbial colonies (hematoxylin and eosin; original magnification ×200).

The surgical specimen, including the extracted teeth, was submitted for routine histopathology (hematoxylin and eosin stain). Histopathologic analysis identified the presence of acellular, avascular bone tissue, with reversal lines, irregular surface, extensive marrow spaces, and Haversian canals. An amorphous material, acellular and slightly basophilic, suggesting microbial colonies, was also observed (Figure 3). The microscopic findings were consistent with a diagnosis of chronic suppurative osteomyelitis.

**Figure 4:**
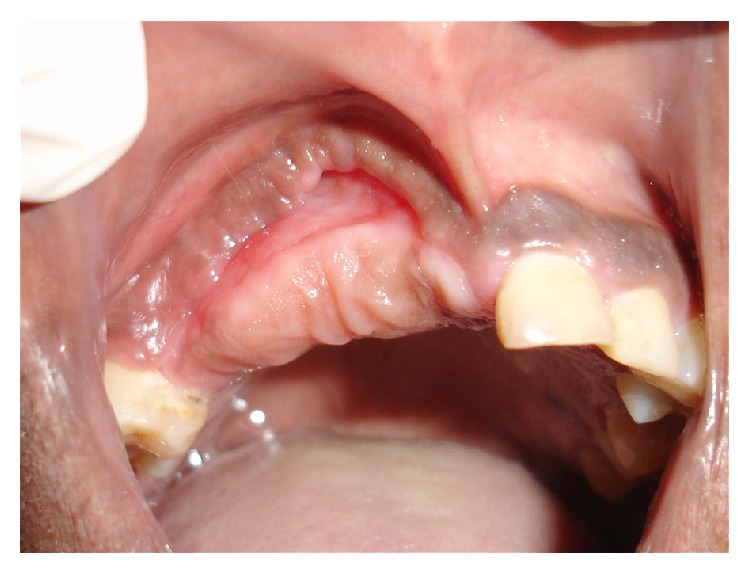
Clinical image at 6-month follow-up. Note complete healing of treated area.

At the first postoperative visit (7 days) the surgical site exhibited partial healing and sutures were removed. The patient was subsequently referred to the integrated clinic for follow-up and for continuation of required additional dental treatment (including restorative work). At the 6-month postoperative visit the surgical site had completely healed and there was no sign of recurrence (Figure 4).

## 3. Discussion

This report presented a case of chronic suppurative osteomyelitis in the anterior maxilla associated with maxillofacial trauma resulting from domestic violence. To the best of our knowledge, this is the first reported case of maxillary osteomyelitis in a context of domestic violence.

The majority of osteomyelitis cases of the jaws involve the mandible while maxillary cases are much less common, due to differences in vascularization [4]. The posterior jaw is most frequently affected, with anterior jaw cases being a rare occurrence [12]. In the present case, the rarely affected anterior maxillary site closely related to the prior trauma from a domestic violence incident. The case presented herein had the typical signs and symptoms of swelling, pain, and draining fistula [14], as well as sequestra and exposed bone [15], and loosening of teeth [13].

Regarding management of the reported maxillary osteomyelitis case, therapy was initiated with oral antibiotics (amoxicillin and metronidazole combination) and followed by surgical removal of the necrotic bone tissue and extraction of associated teeth; this approach achieved good results with no signs of recurrence at the 6-month follow-up. Combination of antibiotic therapy and surgical debridement is effective for the treatment of chronic suppurative osteomyelitis of the jaws [2, 11]. Although surgical debridement eliminates the infected and necrotic bone tissue, it can result in bacteremia [16]; therefore an antibiotic regimen is required. Lesions treated by antibiotic therapy and surgical removal usually do not recur [13]. The infection may recur if the patient suffers new trauma to the involved area or the host response to infection is suppressed [11].

Local and systemic host factors are important in the pathogenesis of osteomyelitis [17]. Osteomyelitis of the jaws is predominantly odontogenic (dental infection related) or traumatic (fracture related) in nature [13, 14]. Trauma is a predisposing factor for osteomyelitis because trauma-related localized tissue thrombosis creates stagnant blood and focal ischemia susceptible to bacterial colonization [18]. Among cases of maxillary osteomyelitis, 10% are caused by trauma [13], due mostly to sports injuries and motor vehicle accidents.

A partially healed fracture and osteomyelitis of the radius in a victim of child abuse have been previously reported [19]; the osteomyelitis was attributed to secondary infection of the inflicted fracture via a hematogenous route [19]. Likewise, in the present case, a maxillary fracture after trauma may have resulted in the secondary development of osteomyelitis. Alternatively, maxillary osteomyelitis may have developed directly after the trauma as a result of scattering odontogenic infection from affected incisor teeth.

Interpersonal physical violence (IPV), which encompasses domestic violence, is a major cause of craniomaxillofacial injury occurring in females aged 15–50 years, with most injuries located in the middle third of the face [20]. Soft tissue injuries (contusions, abrasions, and lacerations) are the most prevalent. Other injuries include epistaxis and fracture or loosening of teeth [21]. Although the probability of suffering facial bone fracture as a result of IPV is low [1], the facial bones can be affected, with mandibular fracture and nose fracture being the most common and maxillary fractures accounting for only 2.4% of such injuries [22].

Most maxillofacial IPV injuries are caused by punching, followed by kicking and slapping [21]. Female victims, mostly married, are usually injured by their spouse, similar to the present case, and have reported at least one previous IPV episode [21]. These data show the relevance of early identification of women with IPV-related injuries. Unfortunately, self-reported IPV injury is low among women exposed to domestic violence [23], hindering the identification of victims. Nonwhite or younger women are more likely to report IPV-related injuries than are white or older women [24]. Halpern et al. [23] have proposed the use of a standard IPV screening questionnaire, the Partner Violence Screen, in cases of suspicious injury. When IPV-related injuries are diagnosed, dentists should introduce preventive interventions to avoid future damage [24].

## 4. Conclusion

Maxillofacial trauma caused by domestic violence can lead to chronic osteomyelitis of the maxilla. The recognition of the injury pattern caused by interpersonal physical violence is important for the diagnosis and management of such cases.
